# Microstructural characterization of corticospinal tract in subacute and chronic stroke patients with distal lesions by means of advanced diffusion MRI

**DOI:** 10.1007/s00234-019-02249-2

**Published:** 2019-07-01

**Authors:** Alfonso Mastropietro, Giovanna Rizzo, Lucia Fontana, Matteo Figini, Bruno Bernardini, Laura Straffi, Simona Marcheselli, Sara Ghirmai, Nunzio Paolo Nuzzi, Maria Luisa Malosio, Marco Grimaldi

**Affiliations:** 1 Institute of Molecular Bioimaging and Physiology, National Research Council, Via Fratelli Cervi, 93, 20090 Segrate (MI), Italy; 2 Institute of Biomedical Technologies, National Research Council, Via Fratelli Cervi, 93, Segrate (MI), 20090 Italy; 3 Neuroradiology Unit, Neuro Center, Humanitas Clinical and Research Center - IRCCS, Via Manzoni, 56, Rozzano (MI), 20089 Italy; 4 Centre for Medical Image Computing, Department of Computer Science, University College London, Malet Place, London, WC1E 7JE UK; 5 Neurorehabilitation Unit, Neuro Center, Humanitas Clinical and Research Center - IRCCS, Via Manzoni, 56, Rozzano (MI), 20089 Italy; 6 Stroke Unit, Neuro Center, Humanitas Clinical and Research Center - IRCCS, Via Manzoni, 56, Rozzano (MI), 20089 Italy; 7 Institute of Neuroscience, National Research Council, Via Vanvitelli, 32, Milan, 20129 Italy; 8 Laboratory of Pharmacology and Brain Pathology, Neuro Center, Humanitas Clinical and Research Center - IRCCS, Via Manzoni, 56, Rozzano (MI), 20089 Italy

**Keywords:** Subacute and chronic stroke, NODDI, DTI, DKI, Corticospinal tract

## Abstract

**Purpose:**

The aim of the paper is to evaluate if advanced dMRI techniques, including diffusion kurtosis imaging (DKI) and neurite orientation dispersion and density imaging (NODDI), could provide novel insights into the subtle microarchitectural modifications occurring in the corticospinal tract (CST) of stroke patients in subacute and chronic phases.

**Methods:**

Seventeen subjects (age 68 ± 11 years) in the subacute phase (14 ± 3 days post-stroke), 10 of whom rescanned in the chronic phase (231 ± 36 days post-stroke), were enrolled. Images were acquired using a 3-T MRI scanner with a two-shell EPI protocol (20 gradient directions, *b* = 700 s/mm^2^, 3 *b* = 0; 64 gradient directions, *b* = 2000 s/mm^2^, 9 *b* = 0). DTI-, DKI-, and NODDI-derived parameters were calculated in the posterior limb of the internal capsule (PLIC) and in the cerebral peduncle (CP).

**Results:**

In the subacute phase, a reduction of FA, AD, and KA values was correlated with an increase of ODI, RD, and AK parameters, in both the ipsilesional PLIC and CP, suggesting that increased fiber dispersion can be the main structural factor. In the chronic phase, a reduction of FA and an increase of ODI persisted in the ipsilesional areas. This was associated with reduced F_ic_ and increased MD, with a concomitant reduction of MK and increase of RD, suggesting that fiber reduction, possibly due to nerve degeneration, could play an important role.

**Conclusions:**

This study shows that advanced dMRI approaches can help elucidate the underpinning architectural modifications occurring in the CST after stroke. Further follow-up studies on bigger cohorts are needed to evaluate if DKI- and NODDI-derived parameters might be proposed as complementary biomarkers of brain microstructural alterations.

## Introduction

Stroke is the second leading cause of death worldwide, after ischemic heart failure (about 6.5 million deaths per year) and the second leading cause of long-term disability in western countries [1, 2]. The main impairment in the aftermath of stroke consists in motor loss, with different kinds of severity ranging from muscle weakness to hemiparesis or hemiplegia, which can affect selectively the upper or lower limbs, or lead to complete paralysis of half of the body. Neuronal death in the brain area affected by ischemia is accompanied and sustained by inflammation, edema, and tissue remodeling, events eventually leading to axonal degeneration of connected brain regions. Among all the white matter tracts that can be directly or indirectly affected by stroke, the corticospinal tract (CST) is the most important one since it constitutes the motor pathway from the cortex to the motor neurons in the spinal cord, and the voluntary motor control of body and limbs rely on its integrity [3].

Magnetic resonance imaging (MRI) techniques are non-invasive diagnostic tools playing a very important role in acute stroke diagnosis by providing useful information for the accurate evaluation of the risks and benefits of interventions and for the prediction of outcomes [4–6]. Given the continuous evolution of the diagnostic imaging field, novel sequences are being implemented and more accurate diagnostic information can be gained from multi-parametric MRI approaches, helping to classify and to date more precisely stroke lesions [7].

Among all available techniques, diffusion MRI, which is sensitive to the random motion of water molecules in tissues, has become an important clinical tool for the diagnosis of brain acute stroke because it is very sensitive to alterations in tissue microstructure [8–10]. Specifically, it has been shown that diffusion tensor imaging (DTI) can detect and quantify CST degeneration after stroke [11], and the reduction of fractional anisotropy (FA), which is a DTI-derived parameter, is also related to the disintegration of axonal structures involved in Wallerian degeneration [12].

Furthermore, DTI parameters were proposed as clinical biomarkers to predict motor recovery and to monitor and foresee the response to neurorehabilitative interventions after stroke [13, 14]. In particular, a strong correlation between FA measured in CST and upper limb motor recovery was described in acute [15], subacute [16], chronic stroke patients [17] and in a longitudinal study [18]. DTI, however, is based on the simplified hypothesis of Gaussian diffusion within the tissues and can be extended by means of diffusion kurtosis imaging (DKI) in order to take into account the non-Gaussian behavior of water diffusion in biologic systems [19]. DKI can add additional information related to the complexity of the microstructural environment of the examined tissue, and it has already been applied to evaluate stroke patients in combination with DTI, showing alterations both in the ischemic lesion and in distant white matter structures [20, 21].

An alternative approach is to fit biophysical models to the diffusion MRI signal, resulting in potentially more specific biomarkers of brain microstructure. A recent example of such kind of models is neurite orientation dispersion and density imaging (NODDI), which can be achieved within clinically feasible scan times [22]. NODDI models the brain architecture with three different compartments: (i) intraneurite volume (F_ic_), modeled as restricted diffusion in sticks; (ii) extra-neurite volume (F_ec_), modeled as hindered, but not restricted anisotropic diffusion; and (iii) a cerebral spinal fluid compartment (F_iso_), modeled as isotropic Gaussian diffusion. The sticks in the intraneurite compartment have multiple orientations within each voxel, and their dispersion is quantified by the orientation dispersion index (ODI), which is sensitive to the dispersion of the fibers composing the tissue both in gray and in white matter.

NODDI has been applied in several clinical conditions such as epilepsy [23], traumatic brain injury [24], brain tumors [25, 26], Alzheimer’s disease [27], Parkinson’s disease [28], and multiple sclerosis [29, 30]. In all these cases NODDI has provided additional information with respect to DTI, thanks to its ability to breakdown tissue architecture into structural elements of the brain tissue [31]. Furthermore, NODDI’s ability to provide information about tissue microstructure has been confirmed by histological findings [29].

NODDI was applied in a few studies with the aim of characterizing brain lesions changes caused by stroke, mainly focusing on the acute [32–34] and subacute phase [35]. More recently, NODDI was applied in a larger cohort of stroke patients in hyperacute, acute, and subacute phases to characterize the lesioned brain regions and exhibited a larger sensitivity compared to DTI and DKI [36]. Furthermore, the orientation dispersion index of NODDI model was found to be predictive of upper extremity motor outcomes 5 weeks after stroke [37].

However, a study on the potential of NODDI in the characterization of the distal microstructural changes induced by stroke in CST has not been performed yet.

In the present paper, we provide results of a study combining DTI, DKI, and NODDI characterization of the post-stroke CST in 17 subjects analyzed at subacute (T_0_ ⁓ 14 days) and chronic (T1 ⁓ 6 months) stages of the pathology. The main goal of this study is to evaluate if the use of advanced dMRI approaches, such as DKI and NODDI, supplementing the classical DTI technique, could provide new insight into the subtle micro-architectural modifications occurring in CST after stroke in both subacute and chronic phases.

## Materials and methods

### Patients selection

Twenty-nine subjects with ischemic stroke in the territories supplied by the middle cerebral artery were enrolled from July 2016 to November 2017. Among them, 17 subjects (age 68 ± 11 years; 9 males and 8 females) were included in this study (10 of them underwent both subacute and chronic evaluations whereas 7 patients were not able to return for the chronic evaluation). Ten of the enrolled subjects could not be included since they did not comply with MRI, despite fulfilling inclusion criteria. Two additional patients underwent MRI but where excluded from the analysis, because the ischemic lesion was localized at the level of the CST and presented additional hemorrhagic infarction. According to clinical guidelines [38–41], among the 17 patients included in the study, 5 underwent intravenous tissue-type plasminogen activator (tPA), 5 fulfilled the criteria for tPA and for endovascular thrombectomy (ET), and 1, who was already under anticoagulant therapy, was subjected to ET only. A detailed description of the subjects’ characteristics is reported in Table 1. The inclusion criteria established for this study were as follows: (i) age 18 years or older; (ii) clinical diagnosis of acute ischemic stroke in the territory supplied by the middle cerebral artery (MCA) causing measurable neurological deficits; (iii) absence of significant comorbidity for neurological pathologies at stroke onset; (iv) absence of inflammatory/infectious pathologies (HIV, HCV, HBV, rheumatoid arthritis, Crohn’s disease) at stroke onset; (v) absence of neoplastic pathologies; (vi) no history of clinically relevant ischemic events (mRANKIN>2); (vii) no classical contraindication to MRI (pregnancy, metallic implants, and claustrophobia). This study was approved by the Humanitas Clinical and Research Center, IRCCS Institutional Ethical Review Board (IERB) (Prot. no. 1550); informed consent was obtained from each participant before inclusion.

**Table 1:** List of patients involved in the study including relevant clinical information. NIHSS = NIH Stroke Scale; ET = endovascular thrombectomy; tPA = tissue-type plasminogen activator

Patient	Sex	Age	Lesion location	Poststroke time (T_0_) (days)	NIHSS at admission SU	Poststroke time (T_1_) (days)	Incidental MRI findings	ET	tPA
1	M	81	Right: Periventricular posterior; splenium corpus callosum paramedianium	15	3	240	Vascular periventricular demyelination	N	N
2	F	76	Left: occipital-parietal and frontal-parietal WM	12	22	210		N	N
3	F	73	Left: frontal-temporal-parietal	12	7	–		N	N
4	M	53	Left: frontal-temporal-insular cortex; temporal-parietal; gyrus angularis;	13	4	204		Y	Y
5	F	48	Right: insular cortex; lenticular nucleus. Corona radiata	17	12	287	Lacunae	N	N
6	M	62	Left: corona radiata; caudate nucleus/putamen; capsula	24	4	282		N	Y
7	M	67	Right: temporo-insular; temporal-parietal; posterior-frontal	15	5	–		Y	Y
8	M	85	Left: Precentral gyrus; premotor-cortex	15	9	274	Chronic cerebral vasculopathy	N	Y
9	F	80	Right: Caudate nucleus/putamen (striatum). Globus pallidus. Temporal lobe uncus. Mesial temporal lobe. Temporal-parietal cortex	13	11	213		Y	Y
10	F	55	Left: temporal-parietal cortex	12	10	–	Lacunae	N	Y
11	F	73	Right: Striatum; capsule; corona radiata	17	5	–		N	N
12	M	67	Right: Caudate nucleus/putamen (striatum)	13	16	187		Y	Y
13	F	65	Right: striatum (caudate nucleus and putamen); premotor cortex; temporal-parietal cortex	8	17	–		Y	N
14	M	55	Left: frontal-temporal-parietal cortex. Corona radiata	11	8	–		N	Y
15	M	79	Left: striatum-capsula; corona radiata	14	5	228	Chronic cerebral vasculopathy	N	N
16	F	76	Left: striatum; capsula; insula	16	19	–		Y	Y
17	M	68	Left: temporo-parietal; temporo-insular; periventricular; fronto-posterior cortex	15	4	189		N	Y
**Mean**		**68**		**14**	**9**	**231**		**6Y**	**10Y**
**Std. dev.**		**11**		**3**	**6**	**36**			

### Neurological functional assessment

Patients admitted to the Stroke Unit (SU) underwent a standard imaging assessment protocol composed of brain CT perfusion and CT angiography to evaluate occlusions and to rule out cerebral hemorrhage. Neurological assessment by the National Institutes of Health Stroke Scale (NIHSS) [42] was applied to objectively evaluate patients’ impairment at admission, at 24 h, 7 days, at dismissal from the SU. Patients were evaluated 24 h post-admission for compliance with inclusion and exclusion criteria indicated in the protocol approved by the IERB and 48 h after admission to the SU they were asked to sign the informed consent for participation in the study.

### MRI acquisitions

Two MRI sessions were proposed in order to evaluate both the subacute and the chronic post-ischemic phase. Seventeen subjects underwent MRI in the subacute phase (T_0_; 14 ± 3 days post-stroke), whereas, in order to evaluate the long term changes in the CST, 10 of them were also scanned at a chronic stage (T_1_; 231 ± 36 days after stroke).

MRI images were acquired using a Siemens MAGNETOM Verio 3-T MRI scanner equipped with a 4 channels phased-array head RF coil. In order to obtain a high-resolution morphological reference, each acquisition protocol consisted of a 3D T_1_-weighted MPRAGE sequence (TR/TE = 1900 ms/2.52 ms, inversion time = 900 ms; NA = 1, flip angle = 9°, slice thickness = 1 mm, slices number = 160, matrix size = 256 × 256, in-plane resolution = 1 × 1 mm^2^; pixel bandwidth = 170 Hz) and a 3D FLAIR sequence (TR/TE = 6000 ms/576 ms, inversion time = 2150 ms; NA = 1, flip angle = 120°, slice thickness = 1.20 mm, slices number = 128, matrix size = 512 × 512, in-plane resolution = 0.5 × 0.5 mm^2^; pixel bandwidth = 781 Hz).

Diffusion-weighted images were acquired using an echo-planar imaging (EPI) sequence (TR/TE = 16,900 ms/96 ms, NA = 1, flip angle = 90°, slice thickness = 2 mm, slices number = 64, matrix size = 128 × 128, in-plane resolution = 2 × 2 mm^2^, pixel bandwidth = 1563 Hz, EPI factor 128).

In order to allow both DTI and NODDI analysis, the diffusion MRI protocol consisted of two shells:

1. Shell 1: 20 gradient directions; *b* = 700 s/mm^2^; 3 *b* = 0 volumes.
2. Shell 2: 64 gradient directions; *b* = 2000 s/mm^2^; 9 *b* = 0 volumes.

The total acquisition time of the whole MRI protocol was about 45 min.

### Image processing and analysis

Diffusion images were corrected for subjects’ motion and Eddy-current-induced distortions with ExploreDTI (http://www.exploredti.com) [43]. The B-matrix was reoriented on DTI data during motion correction [44] and the EPI/susceptibility distortion correction approach was implemented based on elastic registration of b0 images to the corresponding structural image using a b-spline grid sampling [45]. DTI parameters (fractional anisotropy (FA); mean diffusivity (MD); axial diffusivity (AD), and radial diffusivity (RD)) were estimated from both shells combined (*b* = 700 and 2000 s/mm^2^) by using the nonlinear robust estimation of tensors by outlier rejection algorithm implemented in ExploreDTI. DKI parameters (kurtosis anisotropy (KA); mean kurtosis (MK); axial kurtosis (AK), and radial kurtosis (RK)) were estimated from both shells combined (*b* = 700 and 2000 s/mm^2^) by using the linear estimation approach in ExploreDTI.

Furthermore, NODDI parameters (orientation dispersion Index (ODI), intracellular volume fraction (F_ic_) and isotropic fraction (F_iso_)) were calculated using the publicly available MATLAB-based NODDI toolbox (https://www.nitrc.org/projects/noddi_toolbox), which implements the three-compartment model proposed by Zhang et al. [22], based on both the *b* = 700 and *b* = 2000 s/mm^2^ DWI shells.

An expert neuroradiologist (MG) inspected all images from patients in order to exclude that the ischemic event was directly localized in the CST regions that were selected for the analysis. Ischemic lesion volume (ILV) was measured semi-automatically by inspecting volumetric subacute FLAIR scans with the aid of the CT scans obtained during stroke diagnostic assessment. Lesion volumes ranged from 482 to 174,441 mm^3^ (mean ± standard deviation 39,075 ± 48,952 mm^3^).

**Fig. 1:**
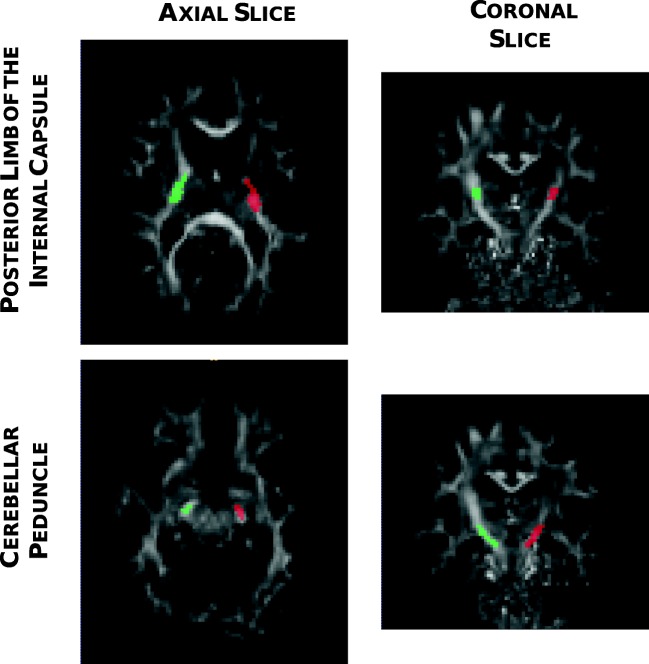
Examples of ROIs outlined at the level of the PLIC (upper panels) and the CP (lower panels) are shown in the axial and coronal planes. The red ROI delineates the ipsilesional hemisphere whereas the green ROI delimits the contralesional hemisphere

For data analysis, an approach based on the definition of a region of interest (ROI) was chosen, focusing on two regions along the CST, the posterior limb of the internal capsule (PLIC) and the cerebral peduncle (CP), as illustrated in Fig. 1.

All maps at T_0_ were co-registered to an MNI-152 standard atlas, and all maps at T1 were realigned to those at T_0_ using an affine linear registration (FLIRT) implemented in FSL [46]. For each subject, ROIs corresponding to PLIC and CP were manually drawn by an expert in MRI imaging (LF) over the FA map in the subacute phase in both ipsilateral and contralateral hemispheres using ITK-SNAP (www.itksnap.org) [47]. ROIs were double-checked by an experienced neuro-radiologist (MG) in order to assure that ROIs were drawn with the correct anatomical localization. For consistency, the same ROIs were used for both T_0_ and T_1_.

For each ROI, mean values and standard deviations were calculated in both the ipsilateral and the contralateral hemispheres.

Statistical differences among groups (ipsilateral vs contralateral; subacute vs chronic) were obtained using a non-parametric two-sided Wilcoxon signed rank test implemented in MATLAB. A *p* value < 0.05 was considered significant. Correlation between ODI and other DTI and DKI parameters was calculated in MATLAB using the Spearman’s correlation method. In order to find some correlation between lesion size and fiber degeneration in both PLIC and CP, we have correlated the ILV with FA, a parameter which is largely used in clinical studies and can be sensitive to Wallerian degeneration [12, 48].

## Results

### Subacute stroke: ipsilateral vs contralateral differences

**Fig. 2:**
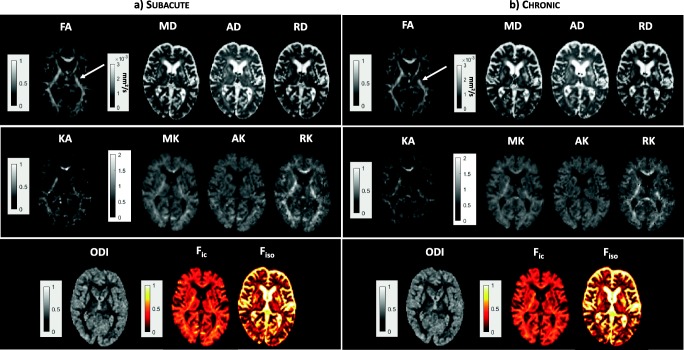
Parametric maps of DTI (FA and MD), NODDI (ODI, F_ic_, F_iso_), and DKI (KA, MK, AK, RK) parameters are shown in the subacute (**a**) and in the chronic (**b**) phase. The area including the PLIC is indicated by a white arrow

**Fig. 3:**
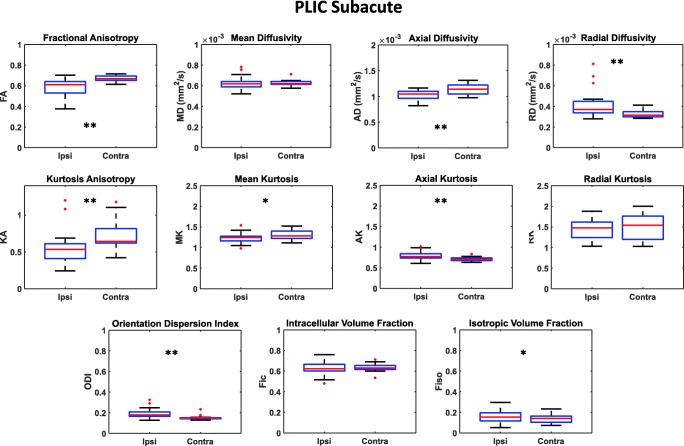
Box plots reporting DTI, DKI, and NODDI parameters calculated for the PLIC in the subacute phase in both the ipsilateral and contralateral hemisphere. Parameters values corresponding to each subject are displayed as red circles. Asterisks refer to the statistical significance of the difference between groups

Figure 2a shows an example of DTI, DKI, and NODDI parametric maps at the level of the ipsilateral and contralateral PLIC regions of the CST, generated from the brain images of stroke patients at the subacute stage. Regarding the PLIC region, the quantitative results of DTI, DKI and NODDI analyses in all patients are shown in fig. 3. As to DTI derived parameters, a significant FA reduction in the ipsilateral hemisphere compared to the contralateral one was measurable (FA_ipsi_ = 0.58 ± 0.09; FA_contra_ = 0.67 ± 0.03; *p* = 0.0008), whereas MD values did not change significantly. The FA reduction was associated to a reduction of AD (AD_ipsi_ = 1 × 10^−3^ ± 8.8 × 10^−5^ mm^2^/s; AD_contra_ = 1.1 × 10^−3^ ± 9.8 × 10^−5^ mm^2^/s; *p* = 0.0008) and an increase of RD (RD_ipsi_ = 4.3 × 10^−4^ ± 1.4 × 10^−4^ mm^2^/s; RD_contra_ = 3.2 × 10^−4^ ± 3.3 × 10^−5^ mm^2^/s; *p* = 0.0012).

Regarding DKI derived parameters, a significant decrease of KA (KA_ipsi_ = 0.57 ± 0.24; KA_contra_ = 0.72 ± 0.19; *p* = 0.0026) and MK (MK_ipsi_ = 1.23 ± 0.13; MK_contra_ = 1.30 ± 0.10; *p* = 0.049) was associated to an increase of AK (AK_ipsi_ = 0.80 ± 0.11; AK_contra_ = 0.71 ± 0.05; *p* = 0.0026).

With respect to NODDI parameters, ODI (ODI_ipsi_ = 0.19 ± 0.05, ODI_contra_ = 0.15 ± 0.02, *p* = 0.0086) and F_iso_ (F_iso-ipsi_ = 0.16 ± 0.06, F_iso-contra_ = 0.14 ± 0.04, *p* = 0.049) were significantly increased in the ipsilateral hemisphere whereas Fic did not show any statistically significant difference between ipsilateral and contralateral hemispheres. A significant correlation was found between FA and ODI in PLIC (both ipsilateral and contralateral) with ρ = − 0.79 and *p* value < 0.0001 (Fig. 8).

**Fig. 4:**
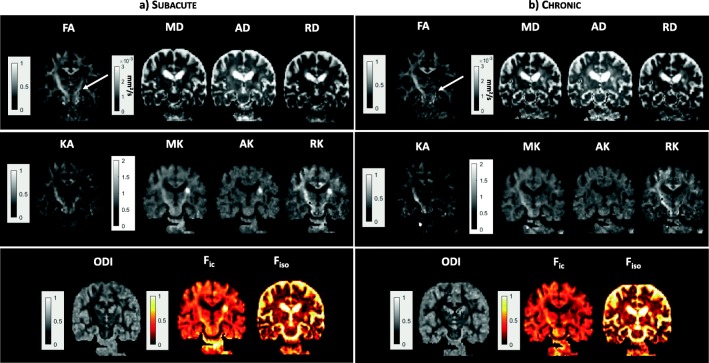
Parametric maps of DTI (FA and MD), NODDI (ODI, F_ic_, F_iso_) and DKI (KA, MK, AK, RK) parameters are shown in the subacute (**a**) and in the chronic (**b**) phase. The area including the CP is indicated by a white arrow

**Fig. 5:**
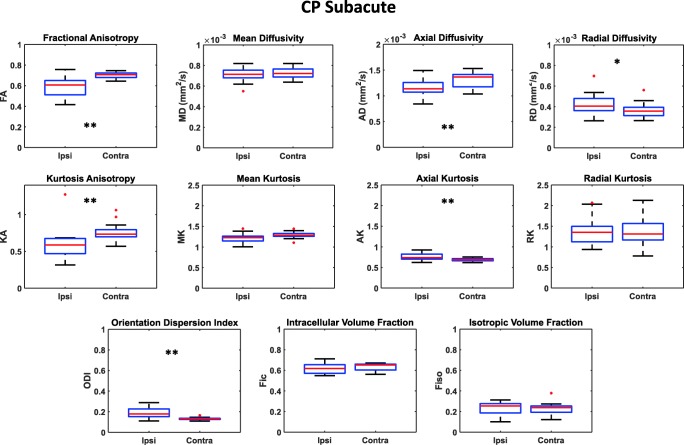
Box plots reporting DTI, DKI, and NODDI parameters calculated for the CP in the subacute phase in both the ipsilateral and contralateral hemisphere. Parameter values corresponding to each subject are displayed as red circles. Asterisks refer to the statistical significance of the difference between groups

An example of DTI and NODDI parametric maps for the CP region in the subacute phase is shown in Fig. 4a. The quantitative DTI, DKI, and NODDI results for CP are shown in Fig. 5. FA values were significantly reduced in the ipsilateral hemisphere compared to the contralateral hemisphere (FA_ipsi_ = 0.59 ± 0.09; FA_contra_ = 0.70 ± 0.03; *p* = 0.001), whereas MD was not significantly different. The FA reduction was associated to a reduction of AD (AD_ipsi_ = 1.2 × 10^−3^ ± 1.5 × 10^−4^ mm^2^/s; AD_contra_ = 1.3 × 10^−3^ ± 1.5*10^−4^ mm^2^/s; *p* = 0.0005) and an increase of RD (RD_ipsi_ = 4.2 × 10^−4^ ± 1 × 10^−4^ mm^2^/s; RD_contra_ = 3.7 × 10^−4^ ± 7.1 × 10^−5^ mm^2^/s; *p* = 0.049).

Regarding DKI-derived parameters, a significant decrease of KA (KA_ipsi_ = 0.58 ± 0.21; KA_contra_ = 0.76 ± 0.12; *p* = 0.0042) was associated to an increase of AK (AK_ipsi_ = 0.76 ± 0.08; AK_contra_ = 0.69 ± 0.04; *p* = 0.0072).

Regarding NODDI parameters, ODI was significantly increased in the ipsilateral CP (ODI_ipsi_ = 0.19 ± 0.04; ODI_contra_ = 0.13 ± 0.01; *p* = 0.0004) whereas all other parameters did not show any statistically significant difference between ipsilateral and contralateral hemisphere (Fig. 5). Also in this case, a significant correlation between FA and ODI values was found (both ipsilateral and contralateral) with ρ = − 0.94 and a *p* value < 0.0001 (Fig. 8). Correlations of ODI with DTI and DKI parameters in subacute phase are listed in Table 2.

**Table 2:** Correlations between ODI and DTI/DKI parameters. In italics are listed the statistically significant correlations

		PLIC				CP			
		Subacute		Chronic		Subacute		Chronic	
		rho	*p* value	rho	*p* value	rho	*p* value	rho	*p* value
DTI	FA	*− 0.79*	*< 0.01*	*− 0.88*	*< 0.01*	*− 0.93*	*< 0.01*	*− 0.93*	*< 0.01*
	MD	− 0.20	0.25	0.25	0.28	− 0.22	0.21	0.36	0.12
	AD	*− 0.64*	*< 0.01*	*− 0.60*	*0.01*	*− 0.59*	*< 0.01*	*− 0.49*	*0.03*
	RD	*0.60*	*< 0.01*	*0.66*	*< 0.01*	*0.56*	*< 0.01*	*0.68*	*< 0.01*
DKI	KA	− 0.34	0.05	*− 0.55*	*0.01*	*− 0.75*	*< 0.01*	*− 0.72*	*< 0.01*
	MK	− 0.20	0.26	*− 0.50*	*0.03*	*− 0.44*	*0.01*	*− 0.57*	*0.01*
	AK	*0.77*	*< 0.01*	*0.49*	*0.03*	*0.85*	*< 0.01*	0.43	0.06
	RK	− 0.14	0.42	− 0.38	0.10	− 0.11	0.55	*− 0.47*	*0.04*

No correlations between ILV and FA were found neither in PLIC nor in CP in the subacute phase.

### Chronic stroke: ipsilateral vs contralateral differences

**Fig. 6:**
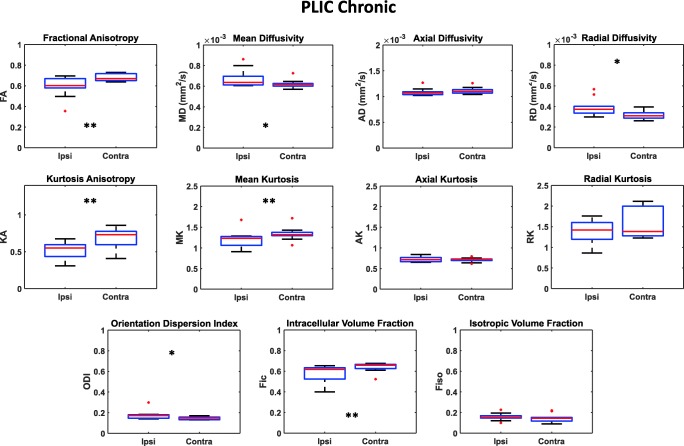
Box plots reporting DTI, DKI, and NODDI parameters calculated for the PLIC in the chronic phase in both the ipsilateral and contralateral hemisphere. Parameters values corresponding to each subject are displayed as red circles. Asterisks refer to the statistical significance of the difference between groups

Figure 2b shows an example of DTI and NODDI parametric maps of the brain in the region of the PLIC region of a stroke patient in the chronic phase. The quantitative DTI, DKI, and NODDI results are shown in Fig. 6. A significant reduction of FA values in the ipsilateral hemisphere compared to the contralateral one (FA_ipsi_ = 0.59 ± 0.10; FA_contra_ = 0.68 ± 0.03; *p* = 0.0098) was confirmed also in the chronic phase. At this time point, MD (MD_ipsi_ = 6.7 × 10^−4^ ± 8.4 × 10^−5^ mm^2^/s; MD_contra_ = 6.2 × 10^−4^ ± 4.0 × 10^−5^ mm^2^/s; *p* = 0.04) and RD (RD_ipsi_ = 3.9 × 10^−4^ ± 8.2 × 10^−5^ mm^2^/s; RD_contra_ = 3.2 × 10^−4^ ± 4.2 × 10^−5^ mm^2^/s; *p* = 0.02) in the lesional hemisphere also increased significantly with respect to the contralateral hemisphere.

As to DKI-derived parameters, a significant decrease of KA (KA_ipsi_ = 0.52 ± 0.12; KA_contra_ = 0.68 ± 0.13; *p* = 0.002) was associated to a decrease of MK (MK_ipsi_ = 1.21 ± 0.20; MK_contra_ = 1.34 ± 0.16; *p* = 0.004) in the ipsilesional PLIC.

Regarding NODDI parameters, in the ROIs of the ipsilateral PLIC of stroke patients, in addition to ODI, persistently being higher (ODI_ipsi_ = 0.18 ± 0.04; ODI_contra_ = 0.15 ± 0.01; *p* = 0.019), F_ic_ was significantly lower (F_ic-ipsi_ = 0.58 ± 0.08; F_ic-contra_ = 0.64 ± 0.04; *p* = 0.0098), whereas F_iso_ did not show any significant difference between ipsilateral and contralateral hemispheres. A significant correlation was found between FA and ODI in PLIC (both ipsilateral and contralateral) with ρ = − 0.88 and *p* value < 0.0001 (Fig. 8).

**Fig. 7:**
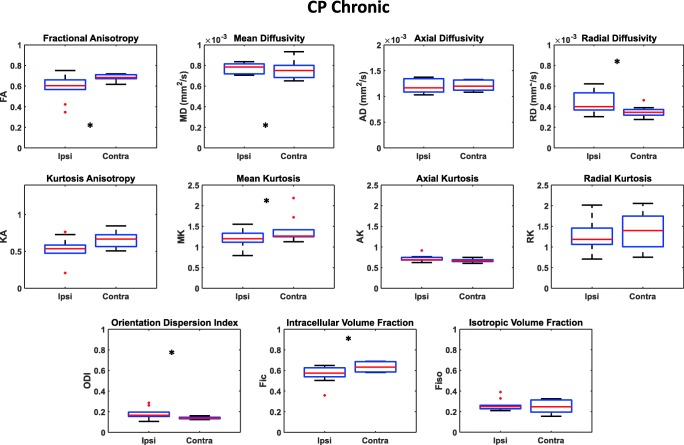
Box plots reporting DTI, DKI, and NODDI parameters calculated for the CP in the chronic phase in both the ipsilateral and contralateral hemisphere. Parameters values corresponding to each subject are displayed as red circles. Asterisks refer to the statistical significance of the difference between groups

Regarding the CP region, Fig. 4b shows an example of DTI, DKI and NODDI parametric maps at chronic stage and Fig. 7 displays the results of the quantitative analysis on all patients. Concerning DTI parameters, FA remained significantly lower (FA_ipsi_ = 0.58 ± 0.11; FA_contra_ = 0.68 ± 0.03; *p* = 0.019) whereas MD (MD_ipsi_ = 8.0 × 10^−4^ ± 1.1 × 10^−4^ mm^2^/s; MD_contra_ = 7.5 × 10^−4^ ± 8.1 × 10^−5^ mm^2^/s; *p* = 0.049) and RD (RD_ipsi_ = 4.4 × 10^−4^ ± 9.4 × 10^−5^ mm^2^/s; RD_contra_ = 3.5 × 10^−4^ ± 4.8 × 10^−5^ mm^2^/s; *p* = 0.049) increased in the ipsilesional hemisphere.

Concerning the DKI-derived parameters, a significant decrease of MK (MK_ipsi_ = 1.21 ± 0.19; MK_contra_ = 1.41 ± 0.30; *p* = 0.019) in the ipsilesional CP was observed.

**Fig. 8:**
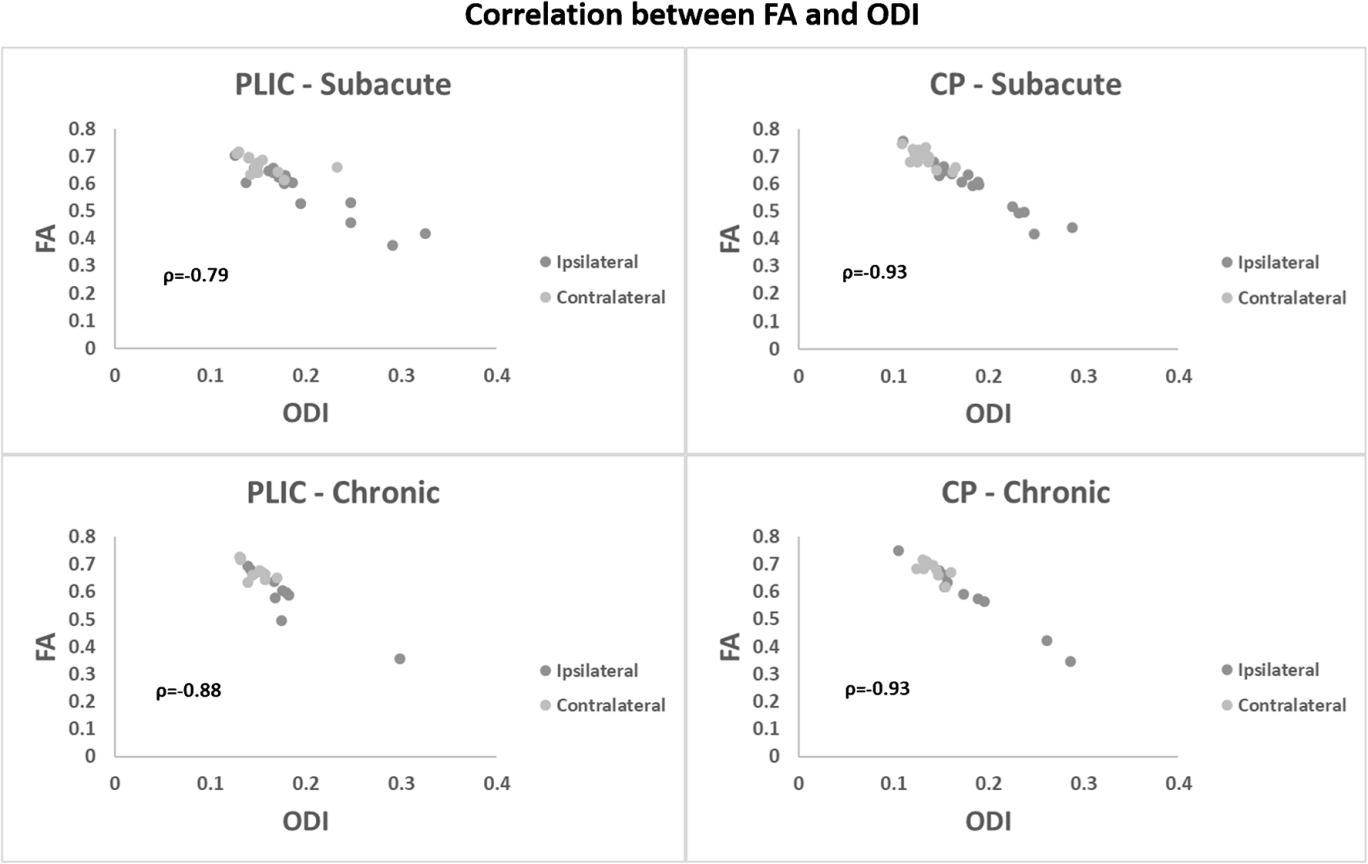
Correlations between FA and ODI parameters evaluated in subacute and chronic phases in both PLIC and CP

The NODDI parameter ODI was persistently higher in the ipsilateral CP ROIs (ODI_ipsi_ = 0.18 ± 0.05; ODI_contra_ = 0.14 ± 0.01; *p* = 0.014), whereas ipsilateral F_ic_ exhibited significantly lower values with respect to the corresponding contralateral region (F_ic-ipsi_ = 0.56 ± 0.08; F_ic-contra_ = 0.63 ± 0.04; *p* = 0.019). F_iso_ was unchanged in both hemispheres. Also, in the case of CP, a significant correlation between FA and ODI values was found with ρ = − 0.93 and a *p* value < 0.0001 (Fig. 8).

DTI- and DKI-derived parameter correlations with ODI are listed in Table 2. No correlations between ILV and FA were found neither in PLIC nor in CP in the chronic phase.

## Discussion

In the present paper, we applied standard and advanced dMRI techniques to find possible correlations between diffusion-weighted metrics and microstructural changes of the CST distal to the ischemic lesion at the level of the PLIC and the CP in stroke patients analyzed with high field MRI (3 T) at two time points, one subacute (14 ± 3 days) and the other one chronic (231 ± 36 days). We show that the DKI approach as well as the NODDI model are valid to complement the classical DTI approach, allowing to gather additional information and providing a better insight into the microstructural changes occurring in the CST after stroke.

In the subacute phase, a reduction of FA and AD, as well as an increase of RD in PLIC and CP on the ipsilateral hemisphere to the lesion was observed in a cohort of 17 stroke patients. The FA alteration was not accompanied by altered MD values in the lesioned hemispheres compared to the contralateral. This can be well explained by the pseudo-normalization of diffusivity parameters that occurs during the subacute phase from day 10 to day 15 [7]. In previous DTI-based studies, the FA reduction had been generally interpreted as caused by the loss of structural integrity of fiber tracts [49] or by Wallerian degeneration affecting white matter downstream of the lesion [12]. However, 15 days after stroke might be a too early time point for Wallerian degeneration to manifest and in any case the microstructural changes underlying reduction of FA had not been fully explained so far.

The investigation of NODDI’s parameters, indicating water proton diffusion according to Gaussian isotropic (F_iso_), intraneurite stick (F_ic_), extra-neurite Gaussian anisotropic modality, and the derived fiber dispersion index (ODI) [22] provides novel insight concerning the structural integrity of the analyzed ROIs in the CST. Interestingly, NODDI parameters obtained from the subacute data analysis show that ODI is significantly higher in PLIC and CP of the ipsilesional hemisphere, whereas no significant alterations in F_ic_ parameter could be measured. These findings suggest that, in subacute phase, fiber dispersion (revealed by ODI) is the main alteration, not yet accompanied by other structural alterations such as Wallerian degeneration or gliosis, since no modification in F_ic_ could be observed.

Furthermore, neurite dispersion measured by ODI showed a significant correlation with FA, as expected, both in PLIC and CP, confirming that the fiber orientation dispersion is an important factor in determining the reduction in FA values [22]. In addition to FA, AD, and RD also exhibit a significant correlation with ODI in all the cases. As to DKI-derived parameters, KA, MK, and AK have shown a significant correlation with NODDI parameters in most of the cases. It is worth noticing that the data points in the chronic phase were fewer than in the subacute phase, so a stronger conclusion about parameters correlation with ODI might be just inferred using the subacute dataset. Taking into account these aspects, FA, AD, RD, and AK might be the only parameters showing consistent correlations with ODI.

The interpretation stated above is well supported by the concomitant reduction of AD and the increase of RD observed in our data in both PLIC and CP. At the same time, the reduction of KA and the increase of AK observed in both PLIC and CP are also in agreement with our interpretation since they can be related to the increase of heterogeneity and/or restriction [20] in the axial direction, and subsequently a decrease of kurtosis anisotropy, due to the augmented dispersion of the fibers. It is worth noticing that ODI, RD, and KA (in PLIC) are the only parameters that maintain their modifications also in the chronic phase; they might be the parameters more related to the augmented dispersion of fibers.

In the chronic stage, FA remained significantly lower whereas RD and ODI stayed higher in the ipsilateral PLIC and CP of patients compared to the contralateral areas. The FA reduction described in this paper confirms previous observations in chronic stroke patients obtained using a constrained spherical deconvolution approach [50]. In addition, we could observe also an increase in MD, which importantly supports, in an independent cohort of patients, what had been previously detected [7].

However, the evolution of the ischemic damage induced further changes in the CST that were measurable by diffusion MRI in the chronic phase in both PLIC and CP. The significant reduction in the NODDI parameters F_ic,_ in the ipsilesional chronic regions, may lead to the speculation that a reduction in the fiber volume occurs in parallel to disorganization of fibers indicated by persistent higher ODI values in the ipsilesional hemisphere. This hypothesis is supported by the reduction of RD and MK observed in both PLIC and CP as well as with the persisting reduction of KA in PLIC. Furthermore, since MD changes were just shown in the chronic phase, the increase of this parameter might be also related to the reduction of fiber volume.

All the previous findings together suggest that the microstructural evolution of CST downstream of the stroke lesion in the chronic phase might consist in white matter (WM) neurite loss, compatible with Wallerian degeneration. The neurite loss seems to be accompanied by a slight reduction of fibers dispersion, and this might explain the unchanged values in FA in subacute vs chronic phase.

The latter findings are very interesting and would benefit from the possibility to investigate these events in an animal model allowing histological confirmation of the NODDI findings.

DTI and DKI parameters measured in PLIC and CP are in reasonable agreement with results found in the literature [12, 21, 51], given the limitations to such a direct comparison due to the use of different acquisition protocols and image analysis methods. As to the NODDI-derived parameters, at the best of our knowledge, our study is the first exploring the PLIC and CP in subacute and chronic stroke patients.

Regarding the ILV, we did not find direct correlation to fiber alterations measured in the CST as shown by FA parameter.

All the above considerations support the use of advanced dMRI techniques as a potentially powerful tool that can help elucidate the underpinning architectural modifications occurring after stroke in the CST and that DKI and NODDI’s parameters could be proposed as specific markers of brain tissue microstructure.

All previous papers, which applied NODDI in subacute stroke patients, had analyzed NODDI parameters at the lesion site and had shown an increase in the fiber orientation dispersion index [35, 36], as well as modifications in densities of intracellular and free water diffusion compartments in the lesioned areas [35]. The increase of F_ic_ parameter was also reported in acute brain stroke and it might be related to astrocytic swelling, causing a reduction in extracellular water component due to anomalous transmembrane diffusion [32]. Increased ODI, observed in acute tissue infarction had been explained by the loss of structural integrity whereas a paradoxical increase of F_ic,_ has been proposed to be due to an increased tortuosity of water diffusion paths caused by cellular swelling, which amplifies intracellular and extracellular diffusion [34]. The same findings were also reported in children affected by acute unilateral MCA territory ischemic stroke [33].

The NODDI results shown in the present paper did not focus on the lesional areas, but instead, the intrinsic microstructural alterations of two districts of the CST, the PLIC, more proximal, and the CP, more distal to the lesion, used as a proxy for motor outcome impairment determined longitudinally after stroke, were characterized.

We are aware that the small number of subjects involved in the present study, especially in the chronic phase time point, could represent a limitation to the statistical power of the results. Furthermore, NODDI includes some strict a priori hypotheses on the diffusivities of the compartments that have been developed for normal brain tissues and have yet to be fully validated. However, ODI has shown a good correlation with histology-derived dispersion measures in both normal and pathological conditions [29, 52].

Even considering the potential bias introduced by the unavoidable simplifications of the mathematical model, the modifications revealed by NODDI, as well as by DKI-derived parameters, are nevertheless in good agreement with the known pathology alterations, which supports their potential value as clinical biomarkers.

## Conclusions

This exploratory and preliminary study shows that advanced dMRI, such as DKI and NODDI, can help elucidate the underpinning architectural modifications occurring after stroke in the CST. In particular, NODDI parameters have shown to offer more coherent data across all the experimental conditions. Nevertheless, further follow-up studies on bigger cohorts are needed to evaluate if NODDI parameters might be proposed as complementary biomarkers of brain microstructural alterations along with DTI parameters.
